# BNE-DETR: Nighttime Pedestrian Detection with Visible Light Sensors via Feature Enhancement and Multi-Scale Fusion

**DOI:** 10.3390/s26010260

**Published:** 2025-12-31

**Authors:** Fu Li, Yan Lu, Ming Zhao, Wangyu Wu

**Affiliations:** 1 School of Internet of Things Engineering, Wuxi University, Wuxi 214105, China; lifu@cwxu.edu.cn (F.L.); 202412492581@nuist.edu.cn (Y.L.); 2 School of Computer Science and Technology, Nanjing University of Information Science and Technology, Nanjing 210044, China; 3 School of Cyber Science and Engineering, Wuxi University, Wuxi 214105, China; 4 School of Computer Science, University of Liverpool, Liverpool L69 3DR, UK; wangyu.wu@liverpool.ac.uk

**Keywords:** nighttime pedestrian detection, RT-DETR, multi-scale, fusion

## Abstract

Pedestrian detection faces significant performance degradation challenges in nighttime visible light environments due to degraded target features, background noise interference, and the coexistence of multi-scale targets. To address this issue, this paper proposes a BNE-DETR model based on an improved RT-DETR. First, we incorporate the lightweight backbone network CSPDarknet and design a Single-head Self-attention with EPGO and Convolutional Gated Linear Unit (SECG) module to replace the bottleneck layer in the original C2f component. By integrating single-head self-attention, the Efficient Prompt Guide Operator (EPGO) dynamic K-selection mechanism, and convolutional gated linear units, it effectively enhances the model’s feature representation capability under low-light conditions. Second, the AIFI-SEFN module, which combines Attention-driven Intra-scale Feature Interaction (AIFI) with a Spatially Enhanced Feedforward Network (SEFN), is constructed to strengthen the extraction of weak details and the fusion of contextual information. Finally, the Mixed Aggregation Network with Star Blocks (MANStar) module utilizes large-kernel convolutions and multi-branch star structures to enhance the representation and fusion of multi-scale pedestrian features. Experiments on the LLVIP dataset demonstrate that our model achieves 1.9%, 2.5%, and 1.9% improvements in Precision, Recall, and mAP50, respectively, compared to RT-DETR-R18, while maintaining low computational complexity (48.7 GFLOPs) and reducing parameters by 20.2%. Cross-dataset experiments further validate the method’s robust performance and generalization capabilities in nighttime pedestrian detection tasks.

## 1. Introduction

Pedestrian detection, as a core task in computer vision, aims to rapidly identify and accurately localize pedestrian targets within images or videos. It has found widespread application in critical scenarios such as security surveillance, industrial inspection, and autonomous driving [1]. Under normal lighting conditions, deep learning-based object detection algorithms have achieved significant progress [2,3]. However, when the scene shifts to low-light environments at night, visible light image quality deteriorates sharply. Prominent challenges arise, including uneven illumination, blurred object contours, significant background noise, and highly variable object scales, posing severe difficulties for pedestrian detection [4,5]. Although hyperspectral image processing demonstrates highly efficient feature enhancement capabilities through multi-band information extraction and fusion under normal illumination [6,7,8,9], its performance degrades under low-light conditions at night due to noise interference and spectral information attenuation. While this provides insights for visible-light single-modality detection, it also highlights its limitations.

Early pedestrian detection methods primarily relied on convolutional neural networks (CNNs) and could be categorized into two major schools: two-stage and single-stage approaches. Two-stage algorithms such as R-CNN [10] and Faster R-CNN [11] gained attention for their high detection accuracy, but their slower processing speeds made them unsuitable for scenarios demanding high real-time performance [12]. With the advancement of deep learning, single-stage algorithms have gradually become mainstream. Among them, the YOLO series [13,14,15,16,17,18,19,20] offers high efficiency by eliminating the need for bounding box proposals and directly predicting both object classification and location. However, these CNN-based detectors remain constrained by their sensitivity to low-light features in nighttime scenarios. For instance, the MDCFVit-YOLO model proposed by Zhang et al. [21] incorporates a ViT module to enhance features for low-light small-object detection [22]. Yet it remains sensitive to nighttime noise, leading to reduced precision for small-scale pedestrians. Furthermore, it lacks adaptive high-frequency enhancement and incurs high computational overhead, making it inefficient for processing multi-scale boundaries with blurred edges. Li et al. [23] introduced the YOLO-FFRD model with a fast fusion module for dynamic small-scale pedestrian detection. However, it suffers from background noise interference under low-light conditions and insufficient feature extraction capability, resulting in high miss rates for distant small-scale pedestrians. Zou et al. [24] improved the YOLOv8-ECSH model for nighttime pedestrian and vehicle detection, utilizing the ECSH module to enhance recall. However, it suffers from insufficient feature relabeling under nighttime noise and relatively static multi-scale fusion, making it difficult to adapt to small pedestrians in shadows [25]. Overall, CNN-based detectors are constrained by their local receptive fields, making it challenging to effectively capture global illumination changes. They are sensitive to noise in complex nighttime scenes and exhibit insufficient feature extraction capabilities [26].

In recent years, detectors centered around Transformers have demonstrated tremendous potential [27,28]. The DETR model proposed by Carion et al. [29] achieves true end-to-end detection by replacing traditional anchor designs with a query mechanism. Zhu et al. [30] further enhanced computational efficiency with Deformable DETR through a deformable attention mechanism. Notably, Baidu’s RT-DETR [31] achieved real-time performance for Transformer-based detectors for the first time while maintaining high precision by introducing an efficient hybrid encoder and an IoU-aware query selection mechanism. However, despite the global modeling capabilities of Transformer architectures, they still face significant limitations in low-light detection tasks at night [32,33]. First, attention mechanisms are sensitive to noise: uneven illumination and background artifacts prevalent in nighttime images easily divert attention resources [34]. For instance, while Huang et al.’s [35] Swin DETR optimizes nighttime detection through local attention, its attention remains susceptible to noise distraction, resulting in low efficiency for small pedestrian detection and high computational overhead. Second, insufficient capture and fusion of fine-grained features: Global attention struggles to perceive locally blurred contours, and existing feature enhancement methods remain inadequate in nighttime scenarios. Li et al. [36] applied FATNet to DETR, enhancing detection through feature attention. However, this mechanism remains sensitive to nighttime artifacts and illumination interference, resulting in insufficient feature interaction, blurred boundaries, and missed detections. It also suffers from slow training convergence and limited multi-scale processing capabilities [37]. Furthermore, computational efficiency and multi-scale adaptability remain critical challenges: while introducing complex mechanisms improves performance, model complexity often increases, hindering deployment. Traditional Transformers also exhibit relatively limited mechanisms for cross-scale feature fusion, making it difficult to dynamically adapt to the drastic scale variations inherent in nighttime scenes [38,39].

To overcome the limitations of single-modal visible light data, multimodal fusion methods have been extensively explored. The DETR-based multimodal fusion approach proposed by Zhao et al. [40] enhances nighttime pedestrian detection performance by integrating visible light and millimeter-wave radar data. Such methods leverage the complementary advantages of different modalities, improving detection effectiveness under low-light conditions to a certain extent. However, they also introduce new challenges: their performance heavily relies on precise pairing and high-quality multimodal data, computational complexity increases significantly, system deployment costs are high, and they struggle to function effectively in mainstream scenarios where only visible light cameras are available. Consequently, due to their strong hardware dependency, system complexity, and limited generalization capabilities, multimodal approaches face significant limitations in universal nighttime visible light pedestrian detection tasks.

Single-modal detection constitutes a core technological branch within nighttime target detection, with thermal imaging modalities demonstrating significant performance potential owing to their inherent physical advantages. This technique generates images by capturing infrared radiation emitted by targets, rendering it entirely independent of illumination conditions. It inherently possesses high target-to-background discrimination capabilities, enabling stable detection without reliance on complex feature enhancement modules. For instance, the lightweight thermal imaging network proposed by Jhong et al. [41], through structural simplification and quantisation optimization, successfully achieved efficient deployment in vehicle-to-everything (V2X) edge scenarios, further validating thermal imaging’s technical feasibility for nighttime tasks. However, thermal imaging solutions face insurmountable engineering bottlenecks: the hardware manufacturing costs of its core sensors far exceed those of visible-light cameras, and its adoption rate remains extremely low in consumer-grade terminals such as standard in-vehicle cameras and civilian surveillance equipment.

Despite the significant engineering value of single-modal visible light detection, its technical challenges far exceed those of thermal imaging solutions. A review of existing research reveals that current visible light nighttime detection methods still face notable bottlenecks: on the one hand, CNN-based detectors are constrained by their local receptive fields, making it difficult to effectively handle global illumination variations in nighttime scenes [42,43], whilst also being sensitive to background noise; On the other hand, whilst the Transformer architecture possesses global modeling capabilities, its attention mechanism is prone to being distracted by illumination artifacts and extraneous light sources within nighttime imagery. This results in inadequate capture of features for blurred, small-scale pedestrians, leading to elevated false-negative rates. Furthermore, while multimodal fusion approaches can enhance performance through cross-modal information complementarity, their effectiveness heavily relies on precise multimodal data pairing. This significantly increases hardware complexity and deployment costs, rendering them unsuitable for mainstream devices equipped solely with visible light sensors. In summary, constructing a detection model under single visible light conditions that balances global and local feature perception, effectively suppresses noise interference, and maintains high computational efficiency has become a critical issue requiring urgent breakthroughs.

Addressing the three core challenges in nighttime visible-light pedestrian detection—weak feature representation, noise interference, and multi-scale adaptation difficulties—this paper proposes the multi-level adaptive model BNE-DETR, using RT-DETR-R18 as the baseline. The core innovation lies in constructing an end-to-end solution tailored for nighttime signal degradation within a lightweight Transformer architecture. This is achieved through a synergistic module design integrating feature enhancement, spatial awareness, and multi-scale fusion. Key contributions are as follows:

1. Introduction of the SECG Feature Enhancement Module: Replacing the C2f bottleneck layer, this module integrates single-head self-attention (enhancing pedestrian contours), dynamic gating (filtering noisy attention), and convolutional gating units (calibrating feature weights). This addresses weak features and noise interference, providing subsequent modules with high signal-to-noise ratio features.
2. Designing the AIFI-SEFN Spatial Awareness Module: Introducing a dual-branch feedforward network within the decoder. Through gated fusion of ‘global attention semantics + original spatial structure’, it prevents distractions from invalid light sources, enhances spatial localisation accuracy for blurred targets, and compensates for the spatial awareness bias inherent in traditional Transformers.
3. Constructing the MANStar Multi-Scale Fusion Module: centered on a star topology, this module combines depth-separable convolutions (reducing computational redundancy at small scales) with gated feature selection (dynamically allocating scale weights). It synergistically adapts to multi-scale variations alongside SECG and SEFN features, resolving the inadequate adaptability of traditional fusion methods to nighttime scale degradation.
4. Model effectiveness is validated across multiple nighttime pedestrian datasets. Experimental results demonstrate that the proposed model outperforms mainstream methods in key metrics, including Precision, Recall, and mAP50, while maintaining low computational complexity and parameter count.

The organizational structure of this paper is as follows: Section 2 reviews the research progress on foundational models and related techniques. Section 3 details the three core modules and the overall network architecture proposed in this paper. Section 4 presents the experimental results and ablation studies for the proposed model and its individual modules, further conducting generalization experiments on several public datasets. Section 5 summarizes the contributions of this research and discusses future work directions.

## 2. Related Work

### 2.1. RT-DETR

**Figure 1:**
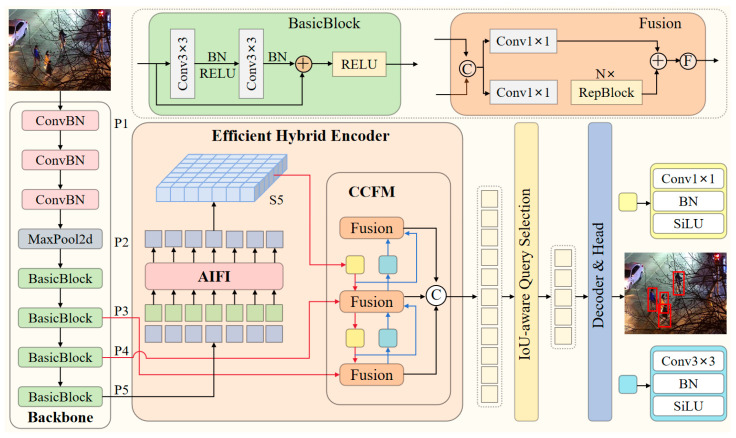
The overall architecture of the original RT-DETR model, comprising the backbone, hybrid encoder, and Transformer decoder with IoU-aware query selection.

RT-DETR is a real-time end-to-end object detection algorithm based on the Transformer architecture proposed by Baidu. Its core advantage lies in eliminating the need for NMS post-processing, effectively balancing detection speed and accuracy. It addresses issues such as high training costs and slow inference in traditional DETR models, while remaining competitive with YOLO. The model structure primarily consists of three components: a backbone network, an efficient hybrid encoder (neck), and a Transformer decoder (head), as shown in Figure 1 [31].

The backbone network employs RT-DETR-R18, which is more suitable for edge deployment compared to larger parameter versions like R34 and R50. Based on ResNet18, it extracts multi-scale features through convolutions and downsampling, feeding only P3, P4, and P5 level features into subsequent layers to reduce computational load and enhance speed [44]. The core innovation lies in the efficient hybrid encoder for the neck region. It processes features using the AIFI mechanism (optimizing P5 features to S5, enhancing intra-scale interactions while reducing complexity) and the CCFM module (fusing cross-scale features via convolution), providing high-quality input to the head encoder. For head detection, a Transformer decoder with an auxiliary prediction head is employed. It utilizes IoU-aware initial query selection and incorporates the DINO denoising strategy to optimize matching and convergence. Through iterative generation of bounding boxes and confidence scores, it enhances detection accuracy and efficiency, enabling real-time detection.

However, when directly applying RT-DETR to nighttime pedestrian detection tasks, its architectural design reveals several inherent limitations. The global attention mechanism in the standard Transformer is highly sensitive to uneven illumination and background artifacts in nighttime images, leading to scattered attention resources. Simultaneously, its feature fusion mechanism struggles to effectively handle the spatial non-uniformity of signal-to-noise ratios characteristic of nighttime scenes, failing to adequately process the discriminative differences between features in illuminated and shadowed regions.

### 2.2. Low-Light Image Processing

The core challenge in nighttime pedestrian detection stems from image quality degradation and target feature attenuation caused by low illumination [45]. Early research primarily focused on image preprocessing techniques, such as decomposition algorithms based on Retinex theory, aiming to reconstruct clear images by estimating illumination and reflection components. However, such preprocessing enhancement methods inherently face uncertainties, struggling to balance detail enhancement with noise suppression. Not only do they readily generate unrealistic artifacts and disrupt feature consistency, but their optimization objectives also remain disconnected from the downstream detection task [46]. To overcome this limitation, the research paradigm has gradually shifted from separate image enhancement to task-oriented joint optimization. Its core idea is to directly construct representations at the feature level that are robust to illumination variations and noise interference. For instance, Li et al. introduced a fast fusion module in the YOLO-FFRD model to enhance features of dynamic, small-scale pedestrians, essentially performing adaptive enhancement of degraded features within the network. Zhang et al. proposed MDCFVit-YOLO, which incorporates a visual Transformer module to model global context, compensating for the limitations of local features in low-light conditions and their sensitivity to noise. These approaches exemplify the advanced concept of “enhancement” and “purification” within the feature domain [47,48].

### 2.3. Attention Mechanism

Attention mechanisms further focus on critical information through dynamic weight allocation, whose principles closely align with the “selective attention” mechanism in human vision, providing significant inspiration for object detection in complex nighttime scenarios [49]. In nighttime environments characterized by scarce photons and significant noise, this mechanism directs computational resources from low signal-to-noise ratio background areas toward potential pedestrian target regions, achieving optimized resource allocation. Inspired by the fovea-periphery division of labor mechanism in the retina, researchers have proposed various non-uniform visual processing models. Shi et al.’s TransNeXt simulates the foveal mechanism through spatially varying processing stages, effectively handling the sparse distribution of nighttime target features while avoiding computational waste on uniformly distributed noise and dark background areas. Chen et al.’s state space model in SEM-Net enhances global context modeling from a continuous spatial dependency perspective, aiding in reconnecting pedestrian contours fragmented by insufficient illumination. Regarding attention mechanism efficiency, Yun et al.’s ShVit demonstrates that single-head attention can achieve robust long-range dependency modeling through streamlined macro-level design. By eliminating redundant computations in traditional multi-head attention, this work provides a viable solution for resource-constrained real-time nighttime detection applications [50]. In recent years, hybrid CNN-Transformer architectures have emerged as a pivotal technological direction for enhancing the efficiency and scene adaptability of attention mechanisms in object detection. For instance, Dai’s CoTNet [51] achieves complementary synergy between local feature modeling and global information exchange by embedding convolutional operations within Transformer blocks, significantly improving detection robustness in noisy environments. Inspired by this, this paper implements global attention modeling through the dynamic sparse single-head self-attention (SHSA) mechanism embedded within the AIFI and SECG modules. Concurrently, 3 × 3 and 7 × 7 convolutions are introduced to perform fine-grained and wide-view local spatial feature extraction and detail refinement, respectively. Their synergistic interaction ultimately achieves a dynamic equilibrium between global context capture and local detail enhancement in low-light nighttime scenarios, effectively circumventing the inherent limitations of pure CNN models with restricted local receptive fields and pure Transformer models prone to noise interference.

### 2.4. Multi-Scale and Multi-Branch

The multi-scale challenges in nighttime pedestrian detection present unique characteristics: target scales vary dramatically, and small-scale pedestrians suffer from significantly lower feature signal-to-noise ratios than larger targets due to insufficient illumination. Traditional feature pyramid networks struggle to adapt to this non-uniform distribution of signal-to-noise ratios in nighttime feature maps through linear fusion of multi-level features, resulting in fused features that fail to achieve optimal detail and semantic representation [52,53]. To overcome the limitations of traditional FPN, researchers have proposed more advanced modeling paradigms from different perspectives. Chen et al. introduced state space models into visual tasks, leveraging their continuous temporal properties to bridge feature discontinuities caused by noise, establish robust long-range dependencies, and effectively integrate ambiguous pedestrian contours. Feng et al. adopted a graph theory approach, employing hypergraph computation to explicitly model higher-order relationships between objects. This structured reasoning capability is crucial for understanding complex spatial relationships common in nighttime scenes, such as severe occlusions and dense crowds. At the architectural level, multi-branch structures and novel convolutional kernels further enhance multi-scale feature processing. Multi-branch architectures explicitly learn feature representations across different scales through parallel pathways, overcoming the limitations of single-path representations. Concurrently, the resurgence of large convolutional kernel designs offers a new technical approach. Their expansive receptive fields integrate distant semantic information, enabling effective inference of the overall contours of ambiguous objects in low-contrast nighttime scenes [54].

### 2.5. Summary of Existing Methods

To clearly define the research gaps and contributions of this study, we have constructed a comparative table (Table 1) for a systematic comparison of BNE-DETR against four representative nighttime low-light pedestrian detection methods, including Transformer-based approaches and multimodal fusion methods. The table encompasses dimensions such as innovation points, modalities, and others. Shortcomings of existing approaches primarily include: noise-sensitive attention mechanisms, inadequate static multi-scale fusion, high computational overhead, and limited robustness to single visible-light modalities. BNE-DETR addresses these gaps through a lightweight hybrid architecture, dynamic gating, and star-shaped fusion.

## 3. Methods

### 3.1. Improved Model BNE-DETR

**Figure 2:**
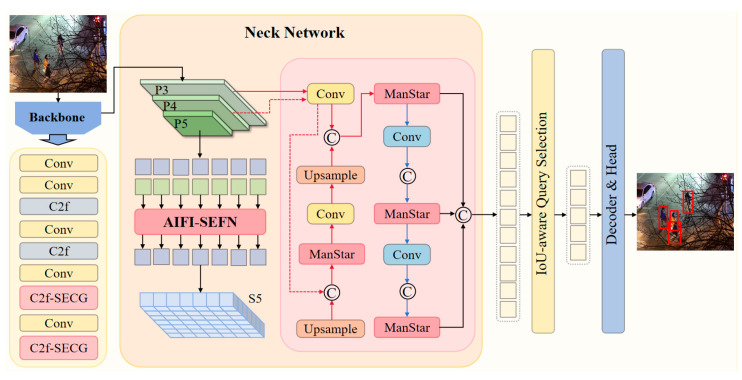
The overall architecture of our proposed BNE-DETR model, highlighting the three core improvement modules: C2f-SECG, AIFI-SEFN, and MANStar.

In this paper, we propose an enhanced RT-DETR model—BNE-DETR—which addresses the unique challenges of nighttime visible-light scenarios by combining feature enhancement mechanisms with multi-scale adaptive fusion. The model comprises three core modules: the Adaptive Screening Enhancement Module (C2f-SECG), the Spatial Enhanced Attention Feedforward Module (AIFI-SEFN), and the Mixed Aggregation Network with Star Blocks (MANStar). While preserving the real-time capability of the RT-DETR-R18 baseline architecture, our model integrates three key enhancement components: C2f-SECG introduces single-head self-attention and dynamic gating mechanisms at the backbone stage, effectively addressing nighttime illumination variations and object degradation through feature purification and contour enhancement; AIFI-SE FN constructs a spatially enhanced feedforward network at the encoder layer. Its dual-path feature selection mechanism focuses computational resources on critical pedestrian regions, significantly improving feature discriminability in noisy environments; MANStar employs a hybrid-aggregated star topology during feature fusion, achieving adaptive representation of multi-scale pedestrian features through multi-branch collaboration and gated selection mechanisms. Through the synergistic interaction of these three modules, BNE-DETR achieves significant detection performance improvements in complex nighttime scenarios, particularly excelling in handling challenges such as uneven illumination, blurred objects, and multi-scale distributions. The improved model architecture is illustrated in Figure 2.

### 3.2. C2f-SECG Module

**Figure 3:**
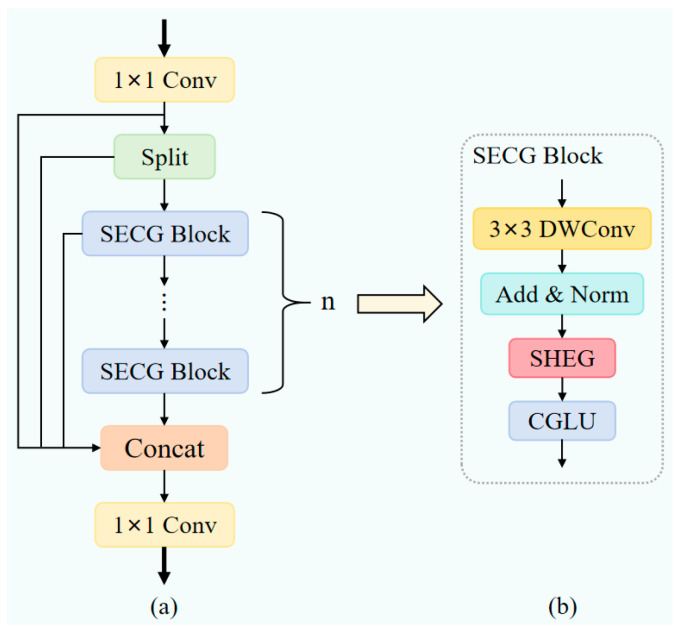
C2f-SECG Module Network Architecture (**a**) C2f-SECG Model (**b**) SECG Block.

To alleviate the insufficient feature representation caused by blurred pedestrian contours and background noise interference in nighttime visible light images, this section designs the C2f-SECG module, as shown in Figure 3. This module replaces the bottleneck layer in C2f with the designed SECG Block. By integrating deep convolutions, incorporating Single-head Self-attention (SHSA [55]) with dynamic gating EPGO [56], and utilizing Convolutional Gated Linear Units (CGLU [57]), it constructs a multi-level, adaptive feature enhancement module that effectively improves the model’s pedestrian feature representation capability under low-light conditions.

The C2f-SECG module adopts a cross-stage partial fusion design philosophy, dividing the input feature stream into two parallel processing paths. One path preserves the original feature stream to maintain gradient flow and critical information, while the other path processes features through a series of our proposed SECG modules. This architecture ensures efficient computation while enabling deep multi-level feature extraction, which is crucial for identifying low-contrast pedestrians against noisy nighttime backgrounds.

The SECG Block, serving as the core processing unit of the C2f-SECG module, employs three key components to achieve adaptive processing of nighttime scenes, as shown in Figure 3b. The module’s input feature map $x\in R^{B\times C\times H\times W}$, where B denotes batch size, C represents the number of channels, and H and W denote the height and width of the feature map, first undergoes a feature preprocessing stage. To address the dual requirements of parameter efficiency and feature retention in nighttime scenes, the module employs a combined strategy of separable convolutions and residual connections [58]:

(1) $$x_{\mathrm{local}}\;=\;x\;+\;\mathrm{BN}(\mathrm{Conv}2d(x,\;\mathrm{groups}\;=\;C))$$

This design significantly reduces the number of parameters while preserving local feature extraction capabilities, effectively preventing overfitting on limited nighttime data. The introduction of residual structures ensures that subtle yet critical pedestrian contour information is transmitted losslessly to deeper layers, preventing the loss of valuable clues during the initial feature transformation stage.

**Figure 4:**
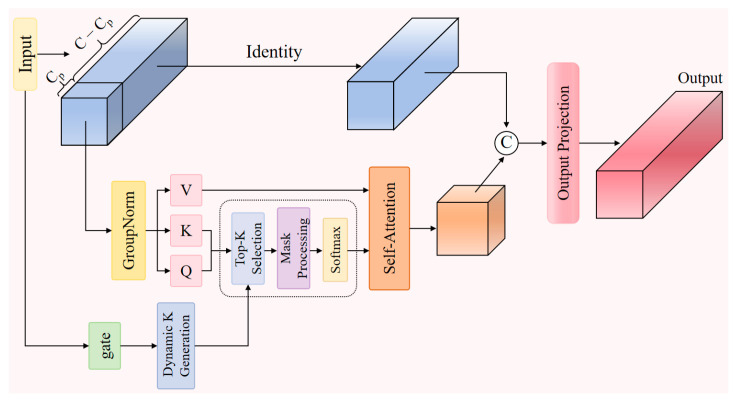
Network Architecture of the SHEG Component.

The preprocessed features enter the core component SHEG, which splits the input features into two parts: one for attention calculation and the other retained in its original state, as shown in Figure 4.

Specifically, the first $C_{p}\;=\;C/4$ channels are used for attention calculations, while the remaining $C\;-{\;C}_{p}$ channels remain the original feature information:

(2) $$x_{1},{\;x}_{2}\;=\;\mathrm{split}(x,\;[C_{p},\;C\;-{\;C}_{p}])$$

This channel partitioning enables the model to leverage attention mechanisms for capturing global context while relying on raw features to maintain precise spatial localization—particularly crucial when pedestrian boundaries become blurred due to insufficient illumination.

To address unstable feature distribution caused by uneven lighting, we apply group normalization to $x_{1}$ in the convolutional layer before projecting features onto the query and key matrices:

(3) $$Q,\;K,\;V\;=\;\mathrm{split}(\mathrm{Conv}2d\_\mathrm{BN}(\mathrm{GroupNorm}(x_{1})),\;[C_{q},\;C_{q},{\;C}_{p}])$$

Setting the query and key dimension $C_{q}$ to a smaller fixed value essentially forces the attention mechanism to focus on the most discriminative components within the noise-dense nighttime features, preventing the dispersion of limited attention resources on irrelevant noise. The core innovation of the SHEG component lies in the design of its dynamic gating optimization mechanism, which directly addresses the uneven light distribution characteristic of nighttime scenes:

(4) $$G(x)\;=\;\sigma ({\mathrm{Conv}}_{1\times 1}(\mathrm{ReLU}({\mathrm{Conv}}_{1\times 1}\left(x)))\right)$$

(5) $$K_{\mathrm{dynamic}}=\max(1,\;\min(N,\;[N\;\cdot \;{\mathrm{gate}}_{\mathrm{mean}}]))$$

The gated network generates spatial weight maps through two 1 × 1 convolutional layers and the Sigmoid activation function σ. Here, ${gate}_{mean}$ represents the average of the gated output across the entire feature map, reflecting the importance level of the current feature. N = H · W denotes the sequence length, while $K_{dynamic}$ is the dynamically selected number k of attention connections based on feature importance. This mechanism adaptively adjusts the attention scope based on local information density, focusing on greater detail in well-lit areas while concentrating on key contours in dim regions.

Subsequently, attention computation employs dynamic sparsity:

(6) $$\mathrm{Attention}\;=\;\mathrm{Softmax}(\mathrm{TopkMask}(\frac{{\mathrm{QK}}^{T}}{\sqrt{d_{k}}},\;k=K_{\mathrm{dynamic}}))$$

The scaling factor $\sqrt{d_{k}}$ prevents vanishing gradients caused by low vector dimensions. Next is the dynamic mask generation process: First, create a zero-filled mask matrix with dimensions B × N × N. Then, the Topk operation identifies the k positions with the highest attention weights, marking these positions as 1 in the mask matrix. Finally, the attention weights at positions masked as 0 are set to negative infinity. This ensures that after the softmax operation, the probabilities at these positions approach zero. The masked attention weights are normalized through the softmax function:

The sparse attention mechanism automatically filters out unimportant regions in nighttime scenes, focusing on key features of pedestrian targets. The sparse attention then aggregates the value vectors, which are concatenated with the unprocessed features along the channel dimension. After SiLU activation and 1 × 1 projection, the final output is obtained. This design effectively reduces computational complexity while preserving the expressive power of the attention mechanism. The specific implementation formula is as follows:

(7) $$x_{\mathrm{temp}}\;=\;V\;\cdot \;\mathrm{Attention}$$

(8) $$x^{\prime }=\mathrm{Proj}(\mathrm{SiLU}(\mathrm{Concat}[x_{\mathrm{temp}},{\;x}_{2}]))$$

**Figure 5:**
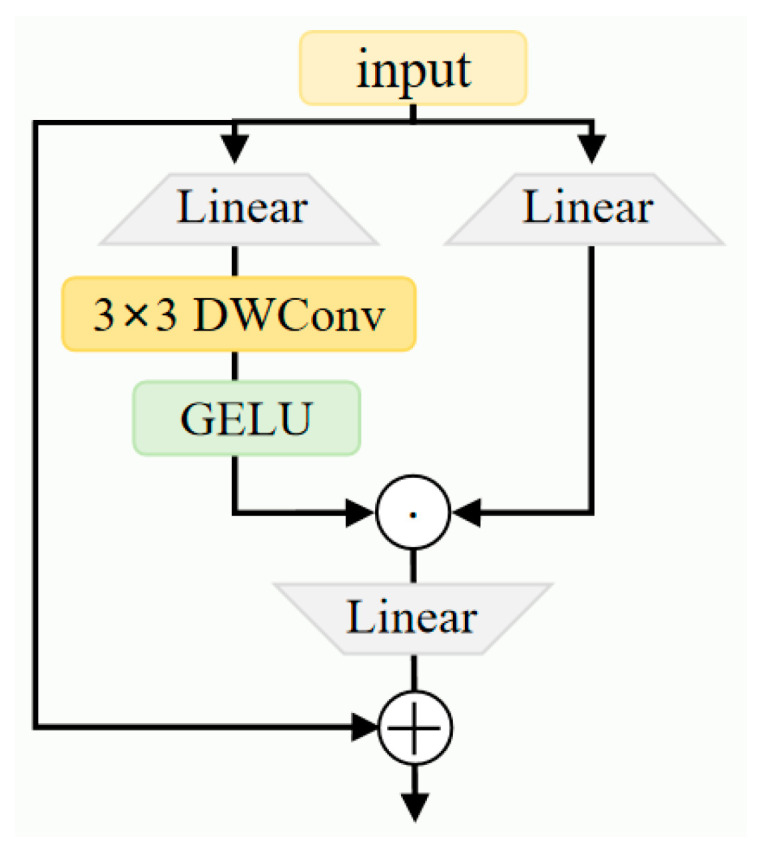
CGLU Architecture Diagram.

Proj represents the projection layer, which effectively fuses the partially processed features from the attention mechanism with the original features to generate the final augmented feature representation. This provides high-quality input for subsequent CGLU processing. The features processed by the attention mechanism then enter the CGLU module, as shown in Figure 5.

The CGLU first performs a linear transformation on the channel dimension via a 1 × 1 convolution, then splits it into two parts:

(9) $$x_{\mathrm{gate}},\;v\;=\;\mathrm{chunk}({\mathrm{Conv}}_{1\times 1}\left(x),\;2\right)$$

Here, $x_{gate}$ denotes the gate signal branch feature, while v represents the value signal branch feature. The gate branch further extracts spatial features through a 3 × 3 depthwise convolution(DWConv) and employs the GELU activation function to enhance nonlinear expressive capability:

(10) $$x_{\mathrm{conv}}\;=\;\mathrm{GELU}({\mathrm{DWConv}}_{3\times 3}(x_{\mathrm{gate}}))$$

Deep convolutions capture local spatial patterns, while the GELU function enhances optimization stability under low-quality image conditions due to its smooth gradient properties. Subsequently, feature selection and weighting are achieved through element-wise multiplication:

(11) $$x_{\mathrm{gated}}\;=\;x_{\mathrm{conv}}⊙v$$

Here, ⊙ denotes element-wise multiplication. This gating mechanism enables the model to dynamically adjust the information flow based on spatial location and feature channel importance, making it particularly suitable for scenarios where targets and backgrounds are poorly distinguished, such as in nighttime environments. Finally, the gated features undergo 1 × 1 convolution projection and are fused with the original input via residual connections:

(12) $$x_{\mathrm{out}}\;=\;x\;+\;{\mathrm{Conv}}_{1\times 1}(x_{\mathrm{gated}})$$

The residual structure not only alleviates the vanishing gradient problem in deep networks but also ensures the complete preservation of original features, providing dual safeguards against potential feature weakening in nighttime images.

The entire C2f-SECG module adheres to the design principle of residual connections:

(13) y = x + CGLU(SHEG(DWConv(x)))

The original input features are combined with the output features processed through all steps to form the final module output.

The C2f-SECG module design incorporates targeted optimisations for nighttime pedestrian detection scenarios: Dynamic Sparse Single-Head Self-Attention (SHSA) captures global contextual information, while 3 × 3 depth convolutions extract local spatial patterns. These components complement each other functionally, effectively enhancing the feature representation of blurred pedestrian contours in nocturnal environments. The gating mechanism selectively transmits effective feature information, significantly reducing interference from nighttime background noise and further enhancing feature discriminability. The Convolutional Gated Linear Unit (CGLU) integrates the local modeling capability of convolutions with the global regulatory properties of the gating mechanism, providing crucial technical support for efficient representation of pedestrian features in low-light environments.

### 3.3. AIFI-SEFN Module

The AIFI-SEFN module addresses the core challenges of inadequate multi-scale feature fusion and complex background interference in nighttime visible-light pedestrian detection. The standard feedforward network in traditional AIFI architectures performs poorly in nighttime scenarios, as its uniform feature processing struggles to handle variations in feature quality caused by uneven illumination and fails to effectively enhance blurred pedestrian contour information [59]. To overcome this limitation, we innovatively replace the standard FFN with the Spatially Enhanced Feedforward Network (SEFN) [60]. Through a dual-branch architecture and a gated fusion mechanism, SEFN enables refined processing of spatial information. This enhancement is particularly well-suited for the feature enhancement demands of low-light nighttime environments.

**Figure 6:**
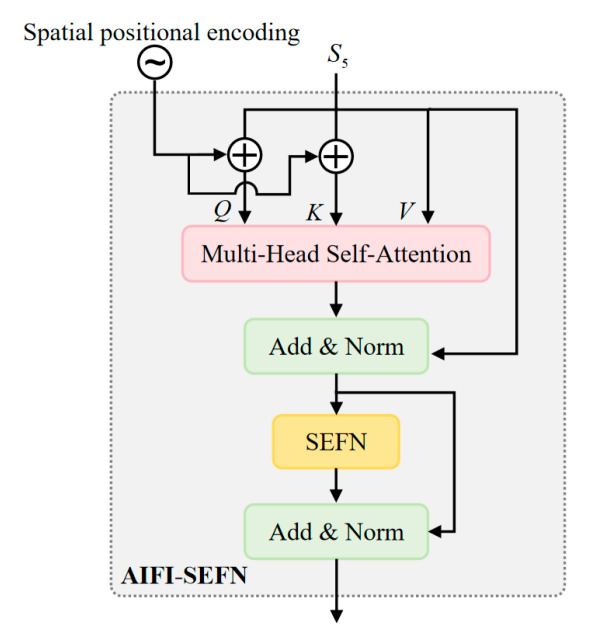
Network Architecture of the AIFI-SEFN Module.

As shown in Figure 6, this module first receives the input feature map from the backbone network. Considering that input features in nighttime scenes often suffer from blurred pedestrian contours and redundant background noise, the module first preserves the original spatial feature map $X_{s}$ to retain complete spatial position information. Subsequently, the feature map is flattened into a [B, H × W, C] sequence format to accommodate the computational requirements of the self-attention mechanism. The feature map undergoes linear projection to generate query (*Q*), key (*K*), and value (*V*) components. However, recognizing the critical role of spatial positional information for target localization in nighttime scenes, the module employs 2D sine-cosine position encoding (PE) to enhance spatial perception capabilities. This encoding is generated via the following formula:

(14) $${\mathrm{PE}}_{\left(\mathrm{pos},2i\right)}\;=\;\sin(\mathrm{pos}/10000^{2i/d_{\mathrm{model}}})$$

(15) $${\mathrm{PE}}_{\left(\mathrm{pos},2i+1\right)}=\cos(\mathrm{pos}/10000^{2i/d_{\mathrm{model}}})$$

Here, pos denotes the position index, i represents the dimension index, and $d_{\mathrm{model}}$ is the feature dimension. By integrating these with *Q* and *K*, respectively, we obtain $Q^{\prime }\;=\;Q\;+\;PE$ and $K^{\prime }\;=\;K\;+\;PE$. This enables the self-attention mechanism to simultaneously consider spatial positional relationships when computing feature similarity, thereby adapting to the modeling requirements of nighttime pedestrian targets across varying positions and scales. The outputs from the multi-head self-attention are concatenated with the original input features via residual connections and undergo layer normalization, ultimately yielding the following formula:

(16) $$X_{\mathrm{attn}}\;=\;\mathrm{LayerNorm}(\mathrm{MultiHeadAttention}(Q^{\prime },K^{\prime },V)\;+\;X)$$

This process effectively alleviates the vanishing gradient problem in deep network training, ensuring the stability of complex feature learning during nighttime.

**Figure 7:**
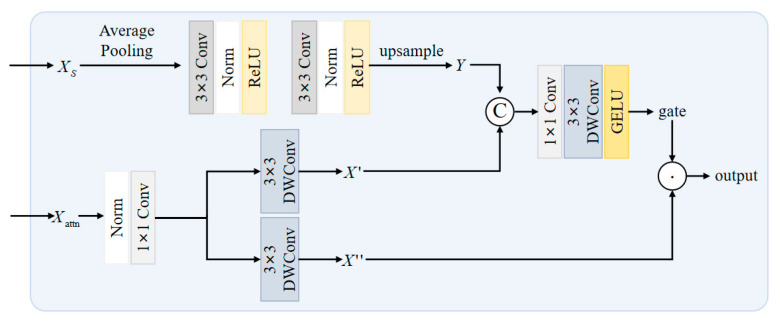
SEFN Spatial Enhanced Feedforward Network Architecture.

Among these, SEFN serves as the core unit of the module, where its dual-branch architecture and gated fusion mechanism are pivotal for achieving spatial information enhancement and effective feature selection (as shown in Figure 7). In Branch one (Attention Feature Processing Branch), the input $X_{attn}$ first undergoes 1 × 1 convolution to expand the channel dimension, enhancing feature expression capacity to accommodate complex nighttime features. Subsequently, deep convolution extracts local spatial correlation information. This operation maintains a 3 × 3 receptive field to capture pedestrian local structures while significantly reducing computational complexity. Finally, channel dimension splitting chunk (2,dim = 1) yields $x_{1}$(X′) and $x_{2}$(X″), which, respectively, serve as the “feature carrier for subsequent fusion” and the “gated base feature”, reserving interfaces for dynamic fusion. Branch Two (Spatial Enhancement Branch) takes the previously saved $X_{s}$ as input. It first downsamples and compresses local noise via average pooling to extract global pedestrian contour information, mitigating interference from nighttime road reflections and light spots. It then undergoes a 3 × 3 convolution for feature transformation. Layer normalization combined with the ReLU activation function enhances feature discriminability. Subsequently, another 3 × 3 convolution and layer normalization deepen the modeling of global spatial correlations. Finally, an upsampling operation restores the features to the same spatial dimensions as Branch One’s output, ensuring precise spatial alignment during the subsequent fusion process.

Building upon this foundation, SEFN achieves adaptive integration of dual-branch features through a gated fusion mechanism. First, branch one’s $x_{1}$ is concatenated with branch two’s upsampled output Y along the channel dimension, forming a composite feature that integrates local attention correlations with global spatial contours. This provides multidimensional discriminative evidence for gated signal generation. The concatenated feature undergoes 1 × 1 convolution to compress redundant channels and consolidate information. Subsequently, 3 × 3 depth convolution further enhances local spatial consistency. Finally, the GELU activation function generates a smooth gating signal. This gating signal undergoes element-wise multiplication with branch one’s $x_{2}$, as expressed in the formula:

(17) $$\mathrm{output}\;=\;\mathrm{GELU}(x_{1})⊙x_{2}$$

To achieve dynamic filtering of effective features. In nighttime scenes, the gated signal assigns high weights to well-lit pedestrian areas and low weights to shadowed or noisy regions, thereby highlighting pedestrian features while suppressing interference.

The SEFN output features undergo 1 × 1 convolutional projection to restore the original channel dimension. They are then connected to $X_{\mathrm{attn}}$ via residual connection and processed through layer normalization to yield the final module output. This process ensures the fused features remain within global contextual constraints while preserving the spatially detailed information enhanced by SEFN. As shown in the formula:

(18) $$X_{\mathrm{out}}\;=\;\mathrm{LayerNorm}({\mathrm{Conv}}_{1\times 1}(\mathrm{SEFN}(X_{\mathrm{attn}},X_{s}))\;+{\;X}_{\mathrm{attn}})$$

The AIFI-SEFN module successfully integrates global spatial contour information with local attention features by introducing a spatial enhancement branch and a gated fusion mechanism. This design enables the model to dynamically filter and enhance beneficial spatial details when encountering uneven nighttime illumination and low-contrast targets. Consequently, it provides the decoder with more discriminative feature representations, effectively improving detection accuracy.

### 3.4. MANStar Module

To address the challenges of varying pedestrian scales and insufficient feature fusion capabilities in pedestrian detection tasks under nighttime visible light conditions, this paper proposes a Mixed Aggregation Network with Star Blocks (MANStar). Building upon the efficient Mixed Aggregation Network (MANet) architecture [38], this module innovatively incorporates Star Feature Extraction Blocks [39]. It aims to enhance the model’s perception and recognition capabilities for pedestrians of varying scales in complex nighttime scenes through multi-branch aggregation and deep spatial feature learning.

**Figure 8:**
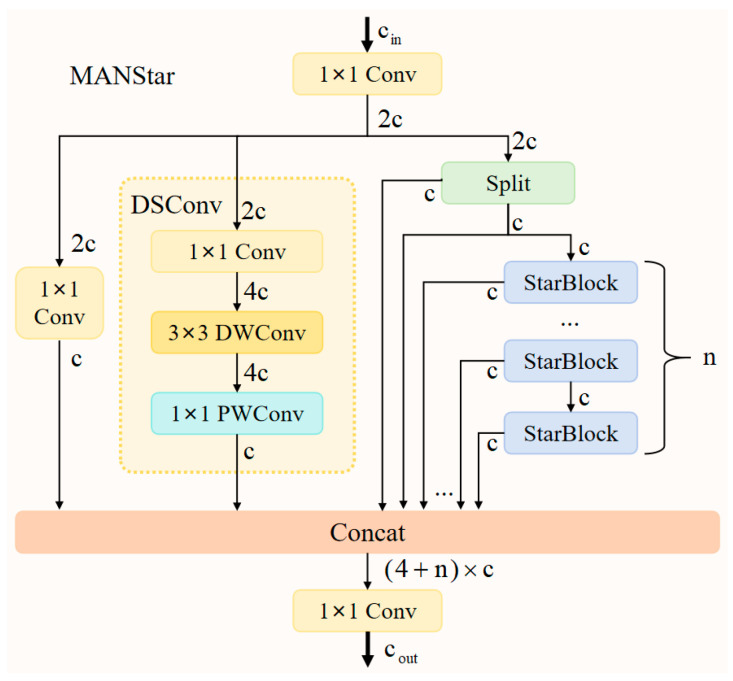
Network Architecture of the MANStar Module.

As shown in Figure 8, the input feature map of the MANStar module has $c_{\mathrm{in}}$ channels, denoted as input features X. First, a 1 × 1 convolutional layer performs channel projection to expand the feature dimension to 2c, laying the foundation for subsequent multi-branch processing. This process can be represented as:

(19) $$X_{\mathrm{mid}}\;=\;{\mathrm{Conv}}_{1\times 1}\left(X\right)$$

After completing the dimensional projection of input features, the 2c-dimensional feature map is fed into a carefully designed four-branch aggregation structure. This architecture comprehensively captures diverse feature representations through four distinct processing paths, providing multidimensional support for subsequent precise modeling of nighttime pedestrian features. It effectively adapts to the complexity and diversity of pedestrian characteristics in nighttime scenarios.

The first path directly extracts fundamental pedestrian contour features through a single 1 × 1 convolution layer while reducing the number of channels to c:

(20) $$X_{1}\;=\;{\mathrm{Conv}}_{1\times 1}(X_{\mathrm{mid}})$$

The second approach employs Deep Separable Convolution (DSConv) for refined processing, comprising three steps: First, a 1 × 1 convolution expands the 2c-dimensional feature channel to 4c, reserving ample channel capacity for subsequent spatial feature extraction; Next, a 3 × 3 depthwise convolution is introduced. While maintaining the 4c channel count, it efficiently captures spatial features with computational complexity significantly lower than standard convolutions. Finally, a 1 × 1 pointwise convolution recompresses the channel count back to c, achieving feature reconstruction and dimensional unification. This approach balances computational efficiency with feature expressiveness while effectively enhancing the discriminative power of local features and improving the accuracy of detail extraction, as detailed below:

(21) $$X_{2}\;=\;\mathrm{DSConv}({\mathrm{Conv}}_{1\times 1}(X_{\mathrm{mid}}))$$

The third approach is the direct splitting method, which equally divides the initial 2c-dimensional features along the channel dimension, yielding two independent c-dimensional feature streams:

(22) $$X_{3},{\;X}_{4}\;=\;\mathrm{Split}(X_{\mathrm{mid}})$$

One of the c-dimensional feature streams is directly fed into the subsequent feature fusion stage (denoted as $X_{3}$), preventing loss of critical original information due to excessive processing.

The fourth path constitutes the Star Block sequence path, which takes another c-dimensional feature stream obtained from segmentation operations as input (denoted as $X_{4}$) and feeds it into a serial processing unit composed of n Star Blocks. Each Star Block strictly maintains consistency between input and output feature dimensions (both c-dimensional). Features are optimized through internal gated feature selection and spatial enhancement via deep convolutions. Unlike traditional designs that utilize only the final sequence output, the output from every Star Block within the sequence participates in subsequent feature fusion. This creates multi-level representations, enabling more effective modeling of complex nighttime pedestrian features. The process is as follows:

(23) $$\begin{array}{l} X_{5}\;=\;{\mathrm{StarBlock}}_{1}\left(X_{4}\right)\;+{\;X}_{4}\\ X_{6}\;={\;\mathrm{StarBlock}}_{2}\left(X_{5}\right)\;+\;X_{5}\\ \ldots \\ X_{4+n}\;={\;\mathrm{StarBlock}}_{n}(X_{3+n})\;+{\;X}_{3+n} \end{array}$$

After completing the processing of the four branches described above, all generated feature streams are concatenated along the channel dimension to form a composite feature map of (4 + *n*) × *c* dimensions. This is ultimately fused and output through a 1 × 1 convolutional layer. This aggregation process can be represented as:

(24) $$X_{\mathrm{out}}\;=\;{\mathrm{Conv}}_{1\times 1}(\mathrm{Concat}(X_{1},{\;X}_{2},\ldots ,{\;X}_{4+n}))$$

This multi-branch, multi-level aggregation strategy ensures the model comprehensively integrates feature information from different processing paths and depth levels, effectively addressing the diversity of pedestrian targets in nighttime scenes.

**Figure 9:**
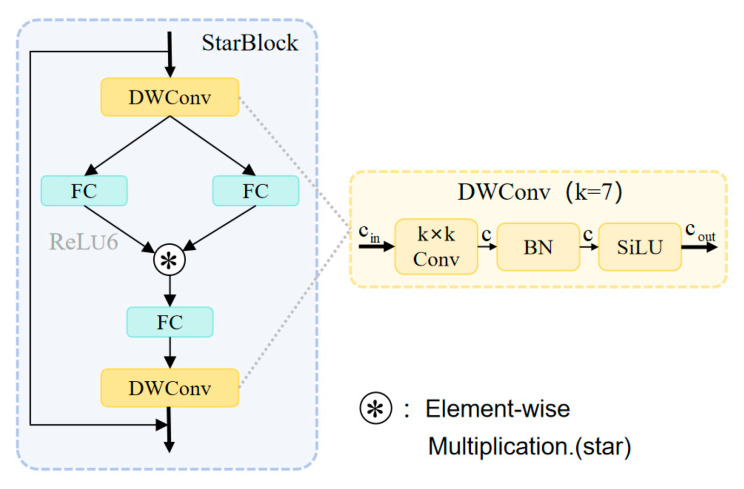
Network Architecture of the Star Block.

The design of Star Block (as shown in Figure 9) draws inspiration from StarNet [24], an architecture that achieves multi-branch coordination through a star topology. However, to address the specific challenges of low-light pedestrian detection at night—such as uneven illumination, blurred targets, and background noise interference—we have modified StarNet. This module achieves effective enhancement of discriminative features by synergistically integrating a dual-branch gated feature selection mechanism with large receptive field deep convolutions. Given input features $X\in R^{B\times C\times H\times W}$, the processing flow is as follows.

Firstly, Star Block combines two applications of large-core deep convolutions (7 × 7) to ensure a broad receptive field captures contextual relationships of blurred nighttime targets. Simultaneously, it integrates a stochastic depth mechanism as a regularization technique, enhancing generalization capabilities in scenes with variable lighting conditions:

(25) $$U\;={\;\mathrm{DWConv}}_{7\times 7}\left(X\right)$$

Subsequently, a dual-branch gated feature selection mechanism is constructed to perform parallel transformation and fusion on U. This mechanism employs two independent 1 × 1 convolutions $f_{1},f_{2}:R^{C}\to R^{rC}$ with non-shared parameters to project features into a higher-dimensional space (where r denotes the channel expansion ratio). It enhances robustness against noise through nonlinear activation and gating operations, dynamically emphasizing information-rich channels while suppressing redundant responses:

(26) $$X_{1},X_{2}\;=\;f_{1}(U),f_{2}\left(U\right)$$

(27) $$M=\mathrm{ReLU}6\left(X_{1}\right)⊙X_{2}$$

Here, ⊙ denotes element-wise multiplication, an operation that dynamically emphasizes information-rich channels while suppressing redundant responses.

To demonstrate the effectiveness of the gating mechanism in high-noise, low-light environments, we conduct a theoretical analysis from the perspective of signal-to-noise ratio (SNR) behavior. Assuming the input feature X comprises useful signal S and noise N (X = S + N). In the dual-branch gating scheme, X_1_ undergoes 1 × 1 convolution and ReLU6 activation to generate the gating signal G(X_1_) ∈ [0, 6]. The saturation property of this activation function ensures that G approaches 0 for low-value noise channels, thereby suppressing noise amplification (G ⊙ N ≈ 0). Conversely, for useful signal channels, G assumes higher values (approaching 6), amplifying the response (G ⊙ S > S). From an SNR perspective, the output feature’s SNR can be approximated as SNR_out ≈ (G ⊙ S)^2^/(G ⊙ N)^2^. G′s noise suppression mitigates risks of weak gradient vanishing or amplified noise. In nighttime pedestrian detection, this adaptive gating ensures gradient preservation for blurred targets while mitigating noise’s impact on multi-scale fusion.

To project features back to the original channel dimension and further refine their spatial structure, the gated output M is first compressed via a 1 × 1 convolution $g:R^{rC}\to R^{C}$, followed by a second large-kernel depthwise convolution to complete spatial refinement:

(28) $$V\;={\;\mathrm{DWConv}}_{7\times 7}\left(g(M)\right)$$

Ultimately, the module output is obtained through residual connections and a random depth mechanism:

(29) Y = X + DropPath(V)

These modifications render the Star Block more suited for nighttime tasks, achieving targeted enhancements in multi-scale fusion and noise suppression compared to the original StarNet. It integrates seamlessly into the MANStar module, forming a hybrid aggregation architecture. When deployed within the deep feature processing path of the MANStar module, its serialized structure preserves the intermediate feature outputs from each Star Block, ultimately achieving hierarchical aggregation at the channel dimension. This design enables multi-granularity synergistic integration—from shallow texture details and mesoscale structural information to deep semantic features—thereby enhancing the model’s multidimensional capabilities for precise capture and feature representation of nocturnal pedestrian targets.

## 4. Results

### 4.1. Experimental Description

#### 4.1.1. Nighttime Pedestrian Detection Dataset

This study utilizes the large-scale visible-infrared paired dataset LLVIP [61], developed by Beijing University of Posts and Telecommunications and specifically designed for low-light vision tasks. The dataset comprises 30,976 images, totaling 15,488 visible-infrared image pairs, primarily captured under low-light conditions with all images rigorously annotated. To accomplish pedestrian detection under low-light conditions at night, this study employs visible-light images from the LLVIP public dataset. Nighttime scene images with a mean gray value < 50 are retained, while daytime and twilight transition frames are excluded, thereby constructing a feature-pure low-light detection subset. Images are filtered based on blurriness, retaining moderately blurred samples to align with the real-world challenges of nighttime imaging. Priority was given to densely populated pedestrian scenes with >3 individuals per frame, alongside samples featuring complex backgrounds such as shadows, noise, and light spots. By amplifying challenging elements within the dataset, its distribution authentically mirrors typical nighttime surveillance challenges. This ensures experimental results remain pertinent to low-light pedestrian detection while enhancing the model’s generalisability in performance evaluation. Images selected from nighttime scenes form a subset comprising 4199 images. These are partitioned according to a 7:2:1 ratio into a training set of 2939 images, a validation set of 840 images, and a test set of 420 images. It is worth noting that this study deliberately employs this subset of visible-light nighttime imagery to strictly focus on the core challenge of monomodal detection using widely available visible-light sensors. Although the dataset is smaller than the complete LLVIP original dataset, this carefully curated subset enables efficient model development and evaluation while precisely aligning with our research objectives. This approach enhances model generalization while reducing training time.

**Figure 10:**
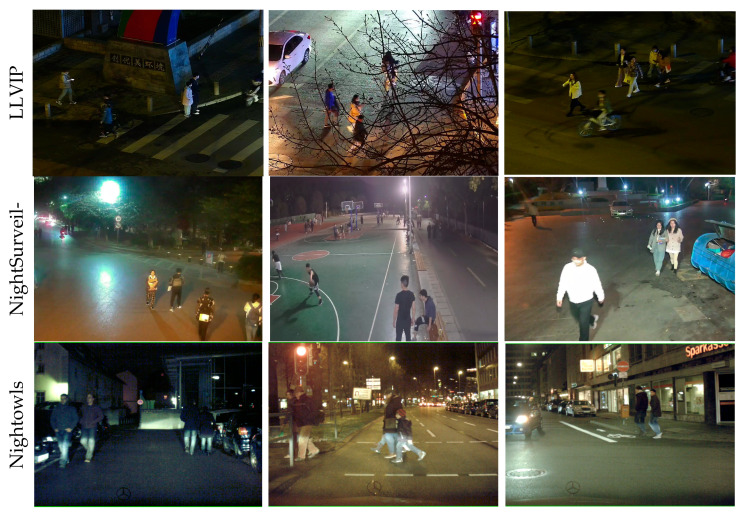
Sample images from the different datasets.

To further validate the proposed model’s effectiveness in nighttime low-light pedestrian detection, experiments were also conducted on two public nighttime pedestrian datasets: NightSurveillance [62] and Nightowls [63]. The NightSurveillance dataset originates from nighttime surveillance videos at 16 intersections, where each video frame was sliced into a single image, yielding 38,113 images. Given that NightSurveillance data primarily consists of captured video frames, adjacent images exhibit high similarity between pedestrian targets and backgrounds. Directly employing this data for training would result in redundancy. We addressed this by extracting one image every three frames, yielding 6351 original images. Subsequently, images featuring dense pedestrian activity and complex background variations were further selected, ultimately constructing a dataset comprising 3525 images. This dataset was split into a training set (2480 images), validation set (719 images), and test set (326 images) at a ratio of 7:2:1. As a large-scale nighttime pedestrian dataset, Nightowls utilized only images from the validation set, similarly selecting one image every four frames to yield 7954 images. This dataset was also partitioned at a 7:2:1 ratio into a training set (5567 images), validation set (1590 images), and test set (797 images). Figure 10 displays sample images from these three datasets.

#### 4.1.2. Experimental Environment and Parameter Settings

This study’s experiments were conducted on Windows 11 using an NVIDIA RTX 4060 (NVIDIA Corporation, Santa Clara, CA, USA) graphics card for model training and evaluation. The PyTorch 2.5 deep learning framework was employed with Python 3.10.16 as the programming language, accelerated by CUDA 12.4 to enhance computational efficiency. Drawing on relevant practical experience, we tailored training parameters accordingly. Input image dimensions were uniformly resized to 640 × 640. The AdamW optimizer was selected, with a base learning rate set to 0.0001 and weight decay coefficient also set to 0.0001. This configuration facilitates stable convergence on nighttime data while preventing overfitting. Training ran for 200 epochs with a batch size of 4. To accommodate low-illumination characteristics of nighttime data, data augmentation strategies were adjusted: mosaic and random augmentation were disabled to reduce noise and artifact interference. Horizontal flipping was applied, and appropriate translation/scaling ratios were set alongside saturation and luminance perturbations in HSV color space to preserve the true distribution of nighttime images. Rectangular inference was employed during validation for efficiency. No pre-trained weights were used in any experiments to ensure fair comparisons. Specific configuration parameters are detailed in Table 2 and Table 3.

#### 4.1.3. Evaluation Indicators

To objectively evaluate the performance of this algorithm model in nighttime pedestrian detection, this paper employs metrics including Precision (P), Recall (R), Mean Average Precision (mAP), model parameter count (Param), and floating-point operations (GFLOPS) to assess the model’s performance. Among these, mAP employs two distinct Intersection over Union (IoU) thresholds: one fixed at 0.5, designated as AP50; the other IoU threshold varies between 0.5 and 0.95, termed AP50-95. Precision (P) measures the extent of false positives, while Recall (R) assesses false negatives. The definitions of Precision and Recall are as follows:

(30) $$P\;=\;\frac{\mathrm{TP}}{\mathrm{TP}\;+\;\mathrm{FP}}$$

(31) $$R=\frac{\mathrm{TP}}{\mathrm{TP}+\mathrm{FN}}$$

TP (true positive) denotes a positive sample predicted as true; FP (false positive) denotes a positive sample predicted as false; FN (false negative) denotes a negative sample predicted as false.

The average Precision (AP) for a single-class label is defined as shown in Equation (32):

(32) $$AP\;=\;\int_{0}^{1}P\left(R\right)\mathrm{dR}$$

mAP denotes the ratio of the sum of the average Precision of all labels to the total number of categories M. The definition of mAP is shown in Equation (33):

(33) $$\mathrm{mAP}\;=\;\frac{\sum_{m=1}^{M}AP}{M}$$

Recall and Precision performance are equally critical. Therefore, this study also employs the F1 score as an additional performance metric. This F1 score represents the harmonic mean of Precision and Recall, effectively balancing both metrics. The F1 score is expressed by Formula (34):

(34) $$F1\;=\;2\;\cdot \;\frac{P\;\cdot \;R}{P\;+\;R}$$

### 4.2. Experimental Analysis

#### 4.2.1. Ablation Experiment of the BNE-DETR Model

In this paper, we randomly selected 420 images from the filtered LLVIP nighttime pedestrian dataset as the test set for the entire experiment. Each proposed module was sequentially added to the RT-DETR model to validate the effectiveness of the proposed modules and their impact on model performance. Table 4 presents the integrated test results for each module, where A, B, and C represent the CSPDarknet, AIFI-SEFN, and MANStar modules optimized via C2f-SECG, respectively.

As shown in Table 4, the ablation study systematically evaluates the contributions of each proposed module. To address the critical question of whether performance gains stem from architectural synergy or simple parameter scaling, we introduce a larger baseline model, RT-DETR-R34 (Exp0), for comparison. Notably, despite employing 31.10 M (compared to the final model’s 15.85 M), the R34 model achieved 89.6% performance relative to R18. However, this remains below BNE-DETR’s 91.5% while using more parameters. This indicates our improvements primarily stem from architectural synergy rather than simple parameter scaling.

**Figure 11:**
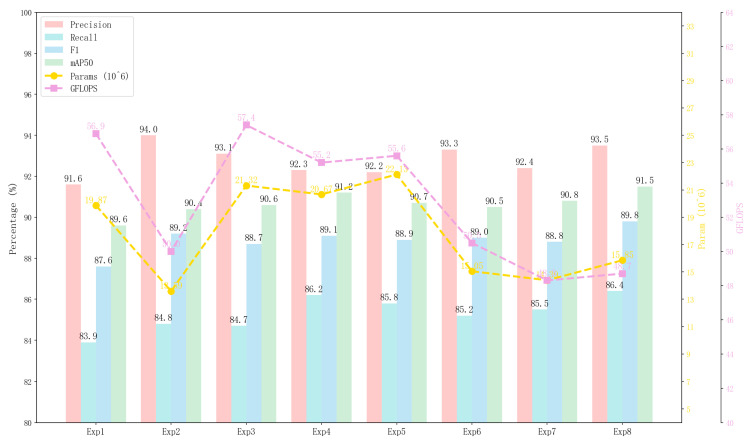
Comparison Results of the Ablation Experiment.

After introducing the C2f-SECG module based on the baseline experiment Exp1, the model’s F1 score improved from 87.6% to 89.2%, while Precision and mAP50 also saw significant increases. Concurrently, the number of parameters and computational complexity decreased. This demonstrates the module’s high effectiveness in enhancing both the model’s overall performance and efficiency. However, the relatively limited improvement in Recall indicates its insufficient capability in identifying missed detection targets. Subsequently, the AIFI-SEFN module improved mAP50 by 1.0%, enhancing spatial feature capture capabilities, but resulted in decreased Precision and increased parameters. Further introduction of the MANStar module boosted mAP50 by 1.6% and Recall by 2.5%, demonstrating the effectiveness of multi-scale fusion, yet similarly faced precision decline and high parameter counts. Among the module combinations, the integration of AIFI-SEFN and MANStar achieved a balanced performance with 92.2% Precision, 85.8% Recall, and 90.7% mAP50, showcasing the complementary strengths of spatial enhancement and multi-scale fusion. Meanwhile, integrating C2f-SECG with AIFI-SEFN maintained a high Precision of 93.3% while limiting parameters to 15.05 M, demonstrating effective synergy between lightweight design and attention mechanisms. Meanwhile, the C2f-SECG and MANStar combination achieved 90.8% mAP50 and 85.5% Recall with only 14.39 M parameters. This setup maintains the advantages of multi-scale fusion while mitigating the Precision drop through its lightweight nature. Both experiments improved select performance metrics while controlling parameter size. The final tri-module integration achieved 91.5% mAP50, 93.5% Precision, 89.8% F1 score, and 86.4% Recall—representing 1.9%, 1.9%, 2.2%, and 2.5% improvements over the baseline, respectively—with a 20.2% reduction in parameters. The reduced GFLOPS consumption also enhances suitability for edge device deployment, achieving optimal overall performance. Figure 11 visually illustrates the comparison results and optimization effects from the ablation experiments.

On the LLVIP subset, we conducted hyperparameter sensitivity analysis on the depth n of Star Blocks, as shown in Table 5. It is worth noting that the dynamic K-selection in the EPGO module is adaptively determined by a lightweight gating network, which does not introduce additional tunable hyperparameters. Therefore, our sensitivity analysis focuses solely on the Star Blocks depth. We varied n from 1 to 5 and observed that mAP50 increased from 0.909 to 0.916, while GFLOPs increased from 44.6 to 52.8, and parameters grew from 14.58 M to 17.12 M. The performance improvement becomes marginal when n exceeds 3, with mAP50 increasing by only 0.1% (from 0.915 to 0.916) as n increases from 3 to 4, while computational cost increases by 4.3%. This demonstrates that further deepening Star Blocks yields diminishing returns in accuracy but significantly increases computational overhead. Balancing performance and complexity, we adopted Star Blocks depth *n* = 3 as the default setting. The stable performance across different depths demonstrates the model’s robustness to architectural design choices.

#### 4.2.2. C2f-SECG Module Analysis

Following the introduction of CSPDarknet, to validate the rationality and effectiveness of the C2f-SECG module design, we conducted systematic ablation experiments on its key submodules. As shown in Table 6 A, B and C represent the SHSA, EPGO, and CGLU submodules, respectively. Experimental results indicate that the original lightweight C2f module significantly reduces the number of parameters while maintaining Precision and Recall, though mAP50 decreases. Building upon this foundation, we progressively introduced each submodule to achieve functional complementarity and performance enhancement. First, the SHSA module was incorporated. Although parameters increased by 0.51 million, mAP50 improved by 1.4 percentage points, laying a solid groundwork for subsequent optimizations. Subsequently, the EPGO module was added. With minimal parameter increment, it further increased mAP50 by 0.3 percentage points while also improving Precision and Recall, demonstrating its sustained enhancement of the model’s overall robustness. Finally, the CGLU module was introduced. While maintaining the total parameter count unchanged, it boosted the model’s Precision by 0.9 percentage points. This indicates that the module, through its gating mechanism, effectively enhances the model’s ability to select discriminative features, focusing on suppressing false positives. When all three modules operate synergistically, C2f-SECG achieves a 1.6 percentage point Precision gain and a 1.9 percentage point mAP50 improvement within a compact parameter range of 13.00 M to 13.59 M. This optimal balance of Precision and efficiency demonstrates outstanding edge deployment value.

Furthermore, to validate the performance of single-head self-attention, this paper conducts a rigorous comparison between C2f + A + B + C and the newly introduced C2f + D + B + C (where D denotes multi-head attention MSMHSA). Both models feature comparable parameter counts (13.59 M vs. 13.73 M) and identical training settings to eliminate confounding factors. Experiments demonstrate that the multi-head attention model achieves results very close to the single-head model in terms of Precision, mAP50, and Recall. This indicates that our EPGO+CGLU design has already ensured semantic discriminative capability in low signal-to-noise ratio nighttime scenes. Simplifying to a single-head model does not compromise robustness; rather, it achieves a better trade-off between parameter efficiency and inference stability.

#### 4.2.3. AIFI-SEFN Module Analysis

To highlight the effectiveness of the SEFN spatial enhancement feedforward network within the AIFI module, we compared it with various feedforward branch architectures and attention mechanisms, including improved approaches such as SEFFN [64], EDFFN [65], and HiLo [66]. As shown in Table 7, AIFI-SEFN achieves significant overall performance improvements solely by enhancing the representation modeling and channel interactions within the feedforward branch, while retaining the original attention mechanism. It achieves 93.1% Precision, 84.7% Recall, 88.7% F1 score, and 90.6% mAP50. Compared to the traditional FFN, SEFN achieves 1.1% and 1.0% improvements in F1 and mAP50, respectively. Compared to SEFFN and DAttention [67], it further enhances F1 and mAP50. Although SEFFN significantly improves Recall, it does so at the cost of reduced Precision. DAttention achieves the highest Precision but suffers from low Recall, increasing the risk of missed detections. Despite increased model parameters, detection robustness is enhanced, particularly for fine-grained and small targets. Overall results demonstrate that, without altering the attention mechanism, strengthening the feedforward structure effectively improves feature quality and translates into end-to-end detection performance gains.

Secondly, we retained the AIFI-SEFN architecture while removing absolute position encoding to generate SEFN-NoPos for comparison. Results indicate that although the CNN backbone possesses implicit spatial modeling capabilities, explicitly injecting 2D cosine position encoding into AIFI-SEFN still consistently enhances detection performance: Precision improved from 0.921 to 0.931, Recall from 0.832 to 0.847, and mAP50 from 0.902 to 0.906. The gains in Recall and overall mAP50 particularly demonstrate that absolute positional encoding enhances the spatial consistency of mid-to-late layer features, enabling query attention to focus more reliably on targets in low-light complex scenes.

#### 4.2.4. MANStar Module Analysis

To validate the superiority of the MANStar module during the feature fusion stage, we conducted an ablation study. As shown in Table 8, the baseline RT-DETR-R18 model achieved 91.6% Precision with the RepC3 module at 19.87 M parameters, but its Recall rate was only 83.9%, indicating significant room for improvement. By incorporating the MANet network, the model saw a slight increase in parameters while achieving a significant 1.7% boost in Recall. The F1 score and mAP50 also improved by 0.5% and 0.3%, respectively. Although Precision decreased by 0.9 percentage points to 90.7%, this phase effectively addressed the baseline’s insufficient Recall. The further optimized MANStar module compressed parameters to 20.67 M while not only boosting Precision back to 92.3% (a 1.6% improvement over MAN) but also increasing Recall by 0.6 percentage points to 86.2%. The final F1 score and mAP50 reached 89.1% and 91.2%, respectively, representing improvements of 1.0% and 1.3% over MAN. This achieved a better balance between Precision and Recall, along with higher overall performance.

### 4.3. Comparative Experiments

#### 4.3.1. Comparative Experiments of Mainstream Models

To validate the effectiveness of the proposed model, we conducted comparative experiments against other mainstream models on the LLVIP dataset. Considering model size, we selected algorithms with comparable parameter counts for comparison, including YOLOv5m, YOLOv8m, YOLOv11m, and RT-DETR-R18. The results are shown in Table 9.

As shown in Table 9, our proposed BNE-DETR model demonstrates superior detection performance across all metrics when compared to mainstream models such as YOLOv5m, YOLOv8m, YOLOv9m, and YOLOv11m. It is noteworthy that to address the evolving landscape of detection architectures, we incorporated the Mamba-YOLO-M model into our comprehensive evaluation. Our model outperforms Mamba-YOLO-M across all core metrics while achieving a 27.3% reduction in parameters and a 93.9% increase in inference speed. Furthermore, even when compared to the larger-parameter YOLOv13l model (27.51 M), our model achieves better performance on all core metrics—Precision, Recall, F1 score, and mAP50 while maintaining only 57.6% of its parameter count and nearly doubling its inference speed (73.5 FPS vs. 36.5 FPS). To contextualize our work within the latest advancements of the DETR family, we compare it with the state-of-the-art RT-DETRv3-R18 model. Although RT-DETRv3-R18 achieves a high mAP50-90 score of 0.498 and boasts the fastest inference speed (84.4 FPS) among the compared DETR models, our BNE-DETR achieves a higher mAP50 score (0.915) with significantly fewer parameters and lower computational cost. This demonstrates outstanding comprehensive capabilities in both accuracy and efficiency. The 73.5 FPS inference speed fully demonstrates its practical deployment potential, far exceeding the 30 FPS requirement for real-time applications. Concurrently, the results validate the RT-DETR architecture series’ advantages over similarly scaled YOLO models in detection tasks. Under challenging detection conditions in low-light complex scenes, our model achieved the best performance among all comparison models with only 15.85 M parameters, while maintaining competitive inference speed. Compared to the original RT-DETR-R18 baseline model, our model reduces parameters by 20.2% while improving mAP50, Precision (P), Recall (R), and F1 score by 1.9%, 1.9%, 2.5%, and 2.2%, respectively, with only a minor trade-off in processing speed. This significantly enhances low-light pedestrian detection capabilities, effectively reducing missed detections while achieving an optimal balance between detection performance and computational efficiency for practical deployment. Although FPS was measured on our research platform, the results clearly demonstrate the model’s real-time processing capability. Comprehensive validation on specific edge devices, such as the NVIDIA Jetson platform, will be addressed in our future deployment-oriented research.

Detecting extremely small pedestrians in low-light nighttime environments poses significant challenges, with baseline models and the proposed model exhibiting generally low average precision (AP) values for small objects, as shown in Table 10. Despite this, BNE-DETR achieves an effective improvement of 0.6 percentage points. Given the extremely low signal-to-noise ratio and sparse small-object features in such scenarios, this improvement holds practical significance. Concurrently, the model’s pronounced performance advantages on medium-to-large-scale objects collectively validate the effectiveness of the SECG, AIFI-SEFN, and MANStar modules, which mitigate nighttime feature degradation and enhance multi-scale feature representation capabilities, particularly for distant and small-sized pedestrians.

#### 4.3.2. Comparison with Two-Stage Method

To validate the effectiveness of the end-to-end enhancement method proposed herein, we compare it with state-of-the-art two-stage pipelines. Specifically, we employed Zero-DCE [68] and SCINet [69] for image preprocessing, followed by detection using the original RT-DETR-R18 model. Results are presented in Table 11. It can be observed that while the two-stage approaches achieve some performance gains (e.g., Zero-DCE+R18 improves mAP50 from 89.6% to 90.5%), these gains are limited and may introduce unrealistic artifacts that degrade Precision. In contrast, our BNE-DETR achieves more substantial performance gains (mAP50: 91.5%) through end-to-end feature-level enhancement. This demonstrates that adaptive enhancement at the feature level holds greater advantages than generic enhancement at the image level.

### 4.4. Visual Experiment

**Figure 12:**
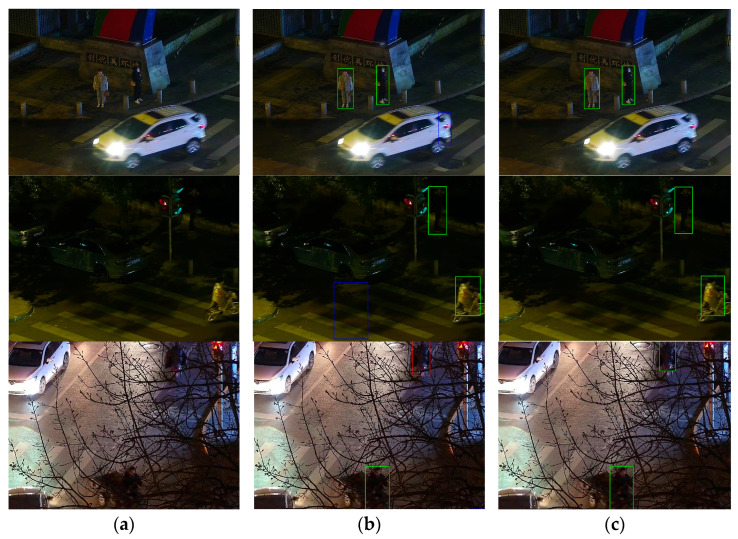
Comparison of Detection Box Effects (**a**) Original (**b**) RT-DETR-R18 (**c**) BNE-DETR; The green boxes indicate correctly detected targets; the blue boxes indicate background elements mistakenly classified as pedestrians; the red boxes indicate actual pedestrians that were not detected.

Figure 12 compares the detection performance of the baseline model RT-DETR-R18 and the proposed BNE-DETR model on the LLVIP nighttime visible light dataset under identical experimental conditions. Green bounding boxes indicate correct detections, blue bounding boxes indicate false positives, and red bounding boxes indicate false negatives. The detection results reveal that the baseline model exhibits significant false positives and false negatives in complex scenarios such as low-light conditions, blurred images, and low contrast—particularly misclassifying noise artifacts as pedestrian targets. In contrast, the proposed BNE-DETR model demonstrates superior detection performance under these challenging conditions, achieving notable improvements in false positive and false negative control. This demonstrates enhanced robustness and practical value.

**Figure 13:**
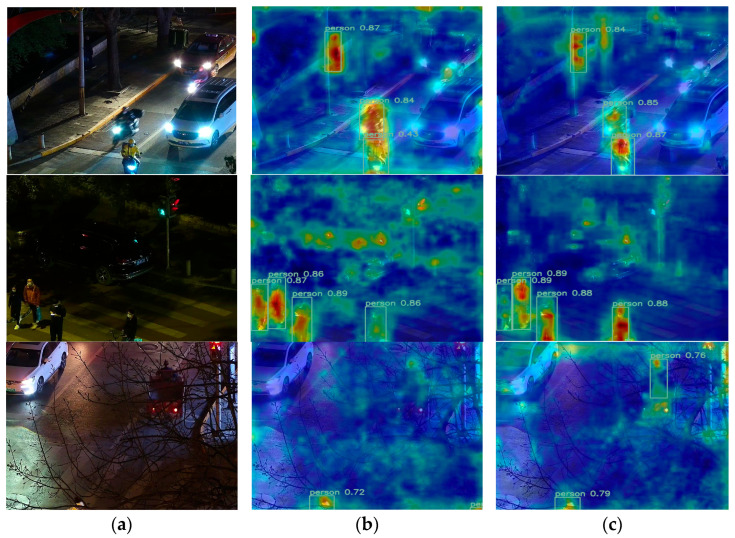
Heatmap Comparison (**a**) Original (**b**) RT-DETR-R18 (**c**) BNE-DETR.

To provide a more intuitive demonstration of the model’s pedestrian detection performance in complex nighttime environments, this paper employs GradCAM++ technology to generate corresponding visualization heatmaps for both the baseline and improved models, as shown in Figure 13. These heatmaps reflect feature point density through varying shades of color, with red denoting the most concentrated regions. To quantitatively validate noise suppression efficacy, we computed the energy concentration ratio: the average activation value within the true bounding box divided by that outside. As shown in Table 12, BNE-DETR achieved an average ratio of 2.7, whereas RT-DETR-R18 attained 1.6. This indicates the improved model exhibits heightened focus on targets while reducing attention to illuminance background noise. Experimental results reveal that RT-DETR-R18 suffers from false positives, false negatives, and positioning inaccuracies when detecting pedestrians under low-light conditions. It also exhibits excessive susceptibility to noise, hindering its ability to focus on irrelevant features. In contrast, the improved model achieves higher detection accuracy by concentrating more precisely on pedestrian targets. These qualitative and quantitative analyses demonstrate that our proposed model achieves superior detection accuracy while mitigating the risks of false positives and false negatives.

**Figure 14:**
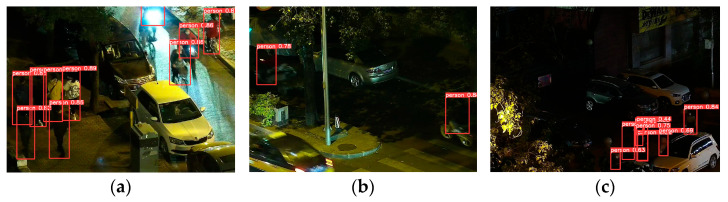
Failure Cases (**a**) Strong point light source glare (**b**) Extreme motion blur (**c**) Small targets with dense occlusions.

Although the BNE-DETR model performs well in most nighttime scenarios, it still exhibits limitations under certain extreme conditions that represent the most critical challenges in real-world nighttime driving. As shown in Figure 14, in scenes with intense point light glare (a), the model may experience missed detections due to sensor saturation and irreversible information loss in over-exposed regions. This phenomenon disrupts the attention mechanism as intense glare amplifies noise gradients and overwhelms subtle pedestrian features. To address this fundamental challenge, future work will explore glare-invariant feature representation learning through adversarial training and develop dedicated pre-processing modules incorporating adaptive brightness normalization for highlight suppression and scene recovery.

For cases with extreme motion blur (b), the core issue is the catastrophic degradation of high-frequency texture and contour information, which compromises temporal continuity and exceeds the model’s feature recovery capabilities from a single frame. This limitation of single-image-based detection motivates a shift towards video-based approaches. Incorporating temporal information through recurrent neural networks (RNNs) or video Transformers would enable video deblurring algorithms and enhance cross-frame consistency for more robust inference.

Furthermore, in scenarios with extremely small targets and dense occlusions (c), while the model responds to some targets, it ultimately fails to successfully decouple and accurately detect all instances. This demonstrates the inherent difficulty of bounding-box regression in ultra-dense scenarios and the low feature signal-to-noise ratio of small, occluded objects. Future improvements will focus on designing specialized detection heads for micro-objects, incorporating multi-scale attention mechanisms, potentially employing center-point or keypoint prediction strategies inspired by crowd counting research. Additionally, graph neural networks could be integrated to explicitly model the spatial relationships between detected proposals, providing structured reasoning capabilities to resolve ambiguities in heavily occluded regions.

These failure cases delineate the adaptive limits of current architectures in dynamic nocturnal environments and point to concrete directions for future research improvements. By integrating physical scene understanding, temporal dynamics, and structured relational reasoning, we aim to build more resilient nighttime pedestrian detection systems capable of handling the full dynamic range of real-world night driving conditions.

### 4.5. Cross-Dataset Generalization Experiment

To validate the detection performance and generalization capability of the BNE-DETR model across different nighttime pedestrian datasets, we conducted further comparative experiments on the NightSurveillance and Nightowls datasets. For the two datasets, we followed an identical 7:2:1 data splitting strategy to independently train and evaluate both the baseline RT-DETR-R18 model and our BNE-DETR model. As shown in Table 13, on the NightSurveillance dataset, all four metrics—Precision, Recall, F1 score, and mAP50—demonstrated significant improvements. This effectively reduced false negatives and false positives in complex nighttime surveillance scenarios. On the NightOwls dataset, Precision slightly decreased from 92.3% to 92.2%, but Recall significantly improved from 86.8% to 89.0%. F1 score increased from 89.5% to 90.6%, and mAP50 rose from 93.6% to 94.5%. This indicates that in dense and small-scale pedestrian scenarios, the model maintains high Precision while further enhancing detection capability. Overall, our model substantially enhances perception and generalization capabilities for weak and low-illuminated targets without sacrificing overall Precision. Even with minor fluctuations in Precision in specific scenarios, these are converted into higher end-to-end detection performance through substantial Recall gains.

## 5. Discussion and Conclusions

Nighttime pedestrian detection is a critical and challenging task in the field of computer vision. Due to complex and variable lighting conditions at night, significant variations in target sizes, and the need to balance real-time detection with stringent edge deployment requirements, achieving high-accuracy, high-efficiency pedestrian detection under low-light and high-noise conditions has become a research hotspot. Timely and accurate identification and localization of pedestrian targets in nighttime environments hold significant practical importance for enhancing the performance of intelligent surveillance systems, ensuring public safety, and reducing traffic accidents.

This paper proposes a lightweight detection model, BNE-DETR, based on the RT-DETR architecture. It aims to systematically address core challenges in pedestrian detection under nighttime visible light conditions, including insufficient accuracy and high rates of false positives and false negatives. While maintaining low computational complexity and parameter count, the model significantly enhances detection performance in complex environments with low illumination and uneven lighting by introducing multi-level feature enhancement and spatial perception mechanisms. Specifically, three core modules are designed: First, the C2f-SECG module, embedded within the lightweight backbone CSPDarknet, fuses single-head self-attention with a dynamic gating mechanism. This effectively enhances the representation of pedestrian contours and edge information while suppressing background noise interference. Second, the AIFI-SEFN module introduces a Spatial Enhancement Feedforward Network within the encoder. Its dual-branch architecture and gated fusion mechanism strengthen the extraction of subtle details, overcoming the susceptibility of traditional attention mechanisms to noise interference in nighttime scenes. Finally, the MANStar module constructs a broad receptive field through its hybrid aggregation architecture and large-core star topology, enabling efficient fusion of multi-scale features. Its gated selection mechanism dynamically focuses on key features, effectively addressing the core challenges of scale variability and background interference in nighttime scenes, significantly enhancing the model’s overall robustness.

Experimental results demonstrate that BNE-DETR achieves significant improvements over the baseline model RT-DETR-R18 on the LLVIP dataset across key metrics, including Precision, Recall, and mAP50. Concurrently, it reduces the number of parameters by 20.2% and lowers GFLOPS requirements, showcasing outstanding lightweight characteristics and potential for edge deployment. Furthermore, cross-dataset generalization experiments on NightSurveillance and NightOwls further validate the model’s robustness and adaptability across diverse nighttime scenarios, particularly excelling in detecting small-scale and low-contrast objects.

This study not only proposes a model that excels in nighttime pedestrian detection tasks but, more importantly, provides a modular design approach for applying lightweight Transformer architectures to complex visual tasks. The concepts embodied by each module—such as “combining global perception with local refinement”, “adaptive feature selection”, and “multi-scale fusion”—exhibit strong transferability and can serve as a reference for other low-quality image analysis tasks.

Although BNE-DETR has made progress in multiple aspects, there remains room for further optimization in the future. We will continue to focus on enhancing model compression and inference speed to meet stricter edge deployment requirements. Simultaneously, addressing more challenging scenarios—such as extreme lighting conditions, adverse weather interference, and object occlusion—particularly the detection of distant small-scale pedestrians and partially occluded targets, will be key priorities for future research. By continuously refining model architecture and training strategies, we aim to further advance the practical application and development of nighttime intelligent surveillance and security systems.

## Figures and Tables

**Table 1:** Summary of Existing Methods.

Model	Key Innovations	Modality	Advantages	Limitations
YOLOv8-ECSH	ECSH	Visible	High recall	Insufficient feature recalibration, static multi-scale
Swin DETR	Local attention optimization	Visible	Optimized for nighttime detection	High computational overhead, noise disperses attention
LSNet	Spatial boosting network, RGB-T fusion	Multi-modal (RGB-T)	Extremely lightweight, high FPS, noise-robust	Sacrifices boundary refinement, relies on thermal modality
WaveNet	Wavelet network + knowledge distillation, RGB-T SOD	Multi-modal (RGB-T)	Preserves edge information, lightweight	Low computational cost but insufficient boundary refinement, relies on thermal
BNE-DETR (Ours)	SECG, AIFI-SEFN MANStar	Visible	Lightweight, noise-robust, high generalization	Needs optimization for extreme glare/blur

**Table 2:** Experimental environment configuration.

Parameters	Settings
Operating systems	Windows 11
GPU	NVIDIA RTX 4060
Deep learning framework	Pytorch 2.5
Accelerated computing framework	CUDA 12.4
Programming Languages	Python 3.10.16

**Table 3:** Experimental parameter configuration.

Parameters	Values
Image size	640 × 640
Epochs	200
Batch size	4
Optimizer	AdamW
Learning rate	0.0001
Decay	0.0001

**Table 4:** Ablation study results of the proposed modules on the LLVIP dataset.

Name	Model	GFLOPS	Param/10^6^	P	R	F1	mAP50	mAP50-95
Exp0	R34	88.8	31.10	0.927	0.833	0.877	0.896	0.492
Exp1	R18	56.9	19.87	0.916	0.839	0.876	0.896	0.491
Exp2	R18+A	50.0	13.59	0.940	0.848	0.892	0.904	0.484
Exp3	R18+B	57.4	21.32	0.931	0.847	0.887	0.906	0.496
Exp4	R18+C	55.2	20.67	0.923	0.862	0.891	0.912	0.487
Exp5	R18+B+C	55.6	22.13	0.922	0.858	0.889	0.907	0.489
Exp6	R18+A+B	50.5	15.05	0.933	0.852	0.890	0.905	0.494
Exp7	R18+A+C	48.3	14.39	0.924	0.855	0.888	0.908	0.493
Exp8	R18+A+B+C	48.7	15.85	0.935	0.864	0.898	0.915	0.496

**Table 5:** Sensitivity analysis of key hyperparameters on LLVIP visible-light subset.

Star Blocks Depth (*n*)	Param/10^6^	GFLOPS	mAP50
1	14.58	44.6	0.909
2	15.21	46.6	0.912
3	15.85	48.7	0.915
4	16.48	50.8	0.916
5	17.12	52.8	0.915

**Table 6:** Ablation experiment on C2f-SECG module.

Model	Param/10^6^	P	R	F1	mAP50
C2f	13.00	0.924	0.835	0.877	0.885
C2f + A	13.51	0.926	0.844	0.883	0.899
C2f + A + B	13.59	0.931	0.848	0.888	0.902
C2f + D + B + C	13.73	0.934	0.851	0.891	0.905
C2f + A + B + C	13.59	0.940	0.848	0.892	0.904

**Table 7:** Comparison experiment on SEFN module.

Model	Param/10^6^	P	R	F1	mAP50	mAP50-95
FFN	19.87	0.916	0.839	0.876	0.896	0.491
HiLo	19.83	0.918	0.858	0.887	0.904	0.493
DAttention	19.87	0.932	0.844	0.886	0.905	0.494
DHSA	20.01	0.917	0.854	0.884	0.906	0.496
SEFFN	19.75	0.912	0.856	0.883	0.903	0.492
EDFFN	19.76	0.928	0.845	0.885	0.901	0.489
SEFN-NoPos	21.32	0.921	0.832	0.887	0.902	0.498
SEFN	21.32	0.931	0.847	0.887	0.906	0.496

**Table 8:** Validity experiment on MANStar module.

Model	Param/10^6^	P	R	F1	mAP50
RepC3	19.87	0.916	0.839	0.876	0.896
MAN	21.96	0.907	0.856	0.881	0.899
MANStar	20.67	0.923	0.862	0.891	0.912

**Table 9:** Detection performance of each model on LLVIP visible light dataset.

Model	GFLOPS	Param/10^6^	P	R	F1	mAP50	mAP50-95	FPS
YOLOv5m	64.0	25.04	0.894	0.812	0.851	0.883	0.473	107.9
YOLOv8m	78.7	25.84	0.894	0.813	0.852	0.882	0.461	91.7
YOLOv9m	76.5	20.01	0.907	0.798	0.849	0.879	0.470	83.0
YOLOv10m	63.4	16.45	0.893	0.791	0.839	0.874	0.464	95.3
YOLOv11m	67.6	20.03	0.894	0.810	0.850	0.881	0.468	90.8
YOLOv12m	67.1	20.10	0.866	0.790	0.826	0.861	0.447	66.7
YOLOv13l	88.1	27.51	0.914	0.812	0.860	0.890	0.465	36.5
Mamba-YOLO-M	49.6	21.80	0.904	0.843	0.872	0.902	0.490	37.9
MDCFVit-YOLO	79.2	20.90	0.918	0.836	0.880	0.904	0.484	58.2
R18 (ours)	56.9	19.87	0.916	0.839	0.876	0.896	0.491	82.7
R34	88.8	31.10	0.927	0.833	0.877	0.896	0.492	67.5
R50	129.5	41.95	0.926	0.852	0.888	0.901	0.494	40.7
RT-DETRv3-R18	60.1	20.00	0.931	0.856	0.892	0.909	0.498	84.4
ours	48.7	15.85	0.935	0.864	0.898	0.915	0.496	73.5

**Table 10:** Performance breakdown by object size on the LLVIP visible-light.

Model	APs	APm	APl
R18	0.168	0.431	0.683
BNE-DETR (ours)	0.174	0.453	0.716

**Table 11:** Comparison with two-stage preprocessing methods.

Method	Pipeline	P	R	F1	mAP50	mAP50-95
R18	Original	0.916	0.839	0.876	0.896	0.491
Zero-DCE + R18	Two-stage	0.926	0.854	0.889	0.905	0.499
SCI + R18	Two-stage	0.930	0.859	0.893	0.911	0.497
BNE-DETR (ours)	End-to-End	0.935	0.864	0.898	0.915	0.496

**Table 12:** Quantitative Analysis of Model Attention Based on GradCAM++ Activation Values.

Model	Average Energy Ratio(In/Out)
R18	1.6
BNE-DETR (ours)	2.7

**Table 13:** Detection performance of the model on different datasets.

Datasets	Model	P	R	F1	mAP50	mAP50-95
LLVIP(ours)	R18	0.916	0.839	0.876	0.896	0.491
	ours	0.935	0.864	0.898	0.915	0.496
NightSurveillance	R18	0.848	0.853	0.862	0.921	0.587
	ours	0.882	0.878	0.880	0.937	0.598
Nightowls	R18	0.923	0.868	0.895	0.936	0.624
	ours	0.922	0.890	0.906	0.945	0.631

## Data Availability

The image dataset subset and core code supporting the findings of this study are publicly available in the GitHub repository “BNE-DETR” at: https://github.com/luy02735-lang/BNE-DETR (accessed on 11 September 2025).
